# Antibiotic therapy for pelvic inflammatory disease: an abridged version of a Cochrane systematic review and meta-analysis of randomised controlled trials

**DOI:** 10.1136/sextrans-2018-053693

**Published:** 2018-10-19

**Authors:** Ricardo F Savaris, Daniele G Fuhrich, Rui V Duarte, Sebastian Franik, Jonathan D C Ross

**Affiliations:** 1 Ginecologia e Obstetricia, Universidade Federal do Rio Grande do Sul-FAMED, Porto Alegre, Brazil; 2 Liverpool Reviews and Implementation Group, University of Liverpool, Liverpool, UK; 3 Department of Gynaecology and Obstetrics, Münster University Hospital, Münster, Germany; 4 Whittall Street Clinic, University Hospital Birmingham NHS Foundation Trust, Birmingham, UK

**Keywords:** antibiotics, pelvic inflammatory disease, systematic reviews, meta-analysis

## Abstract

**Objective:**

To assess the effectiveness and safety of antibiotic regimens used to treat pelvic inflammatory disease (PID).

**Design:**

This is a systematic review and meta-analysis of randomised controlled trials (RCTs). Risk of bias was assessed using the criteria outlined in the Cochrane guidelines. Quality of evidence was assessed using the Grading of Recommendations Assessment, Development and Evaluation.

**Data sources:**

Eight electronic databases were searched from date of inception up to July 2016. Database searches were complemented by screening of reference lists of relevant studies, trial registers, conference proceeding abstracts and grey literature.

**Eligibility criteria:**

RCTs comparing the use of antibiotics with placebo or other antibiotics for the treatment of PID in women of reproductive age, either as inpatient or outpatient treatment.

**Results:**

We included 37 RCTs (6348 women). The quality of evidence ranged from very low to high, the main limitations being serious risk of bias (due to poor reporting of study methods and lack of blinding), serious inconsistency and serious imprecision. There was no clear evidence of a difference in the rates of cure for mild-moderate or for severe PID for the comparisons of azithromycin versus doxycycline, quinolone versus cephalosporin, nitroimidazole versus no use of nitroimidazole, clindamycin plus aminoglycoside versus quinolone, or clindamycin plus aminoglycoside versus cephalosporin. No clear evidence of a difference between regimens in antibiotic-related adverse events leading to discontinuation of therapy was observed.

**Conclusions:**

We found no conclusive evidence that one regimen of antibiotics was safer or more effective than any other for the treatment of PID, and there was no clear evidence for the use of nitroimidazoles (metronidazole) compared with the use of other drugs with activity against anaerobes. More evidence is needed to assess treatments for women with PID, particularly comparing regimens with or without the addition of nitroimidazoles and the efficacy of azithromycin compared with doxycycline.

## Introduction

Pelvic inflammatory disease (PID) in women is described as an inflammation of the upper genital tract and surrounding structures as a result of ascending infection from the lower genital tract—bacteria spread directly from the cervix to the endometrium and on to the upper genital tract.^1^ The signs and symptoms of PID are not specific and may range from asymptomatic to abdominal pain/tenderness, fever, vomiting, dyspareunia and menorrhagia.^2^

The incidence of PID in the UK has been estimated to range between 1.1% and 1.7% among women between 16 and 46 years old.^3 4^ Among women with PID, 10%–20% may become infertile, 40% will develop chronic pelvic pain, and 10% of those who conceive will have an ectopic pregnancy.^5–8^ The cost of pelvic infection has been estimated to exceed US$2.4 billion in the USA, and the mean total cost per episode managed as an outpatient is around US$700.^9^ In the UK, the mean cost of an uncomplicated episode of PID is £163, considering an average of two outpatient appointments per woman across all settings.^10^

PID requires effective treatment to reduce the incidence of chronic pelvic pain, infertility and ectopic pregnancy. The main intervention for treating acute PID is the use of broad-spectrum antibiotics which cover *Chlamydia trachomatis*, *Neisseria gonorrhoeae* and anaerobic bacteria, but the optimal treatment strategy is unclear. A variety of antibiotic regimens and routes of administration (intravenous, intramuscular or oral) have been used, with marked geographical variation. Current practice generally involves the use of multiple agents to provide broad antimicrobial cover, but the best combination of agents is unknown. Guidelines have been produced in the USA,^2^ and in Europe,^11^ to guide therapy, but these have not been based on a systematic review. The authors of the current Centers for Disease Control and Prevention (CDC) and International Union against Sexually Transmitted Infections (IUSTI) guidelines state that there is limited evidence for the need to eradicate anaerobes or for the use of alternative regimens, such as azithromycin.^2 11^

The current review is an abridged version of a Cochrane systematic review and presents the main findings from the primary outcomes and an enhanced discussion section.^12^ This review addresses clinical questions raised in the current guidelines on the treatment of PID^2 11^ regarding the effectiveness and safety of nitroimidazole, the relative benefits of azithromycin versus doxycycline, the use of quinolones, and the relative benefits of cephalosporins compared with clindamycin plus aminoglycoside, to inform future guideline development and clinical practice.

## Methods

We used Cochrane methodology,^13^ following our published protocol.^14^

### Methods for identification of studies

We searched the Cochrane Sexually Transmitted Infections Review Group’s Specialised Register, the Cochrane Central Register of Controlled Trials, MEDLINE, MEDLINE In-Process & Other Non-Indexed Citations, MEDLINE Daily Update, Embase, LILACS and Web of Science up to July 2016. The complete search strategy is available in the Cochrane review.^12^ We screened the reference lists of all identified randomised controlled trials (RCTs) and previous systematic reviews on similar topics for additional relevant articles. Furthermore, we searched trial registers, conference proceeding abstracts and grey literature. We contacted the authors of all RCTs identified by other methods, as well as pharmaceutical companies producing ‘antibiotic therapy’ for ‘pelvic inflammatory disease (PID)’.

### Types of studies

We included RCTs, including those which did not describe their method of randomisation (ie, where the authors stated that treatment was randomised without providing further details). Trials were included irrespective of publication status, publication year or language. We excluded quasi-randomised trials because they produce effects estimates indicating more extreme benefits when compared with RCTs.^13^ We also excluded cross-over and cluster trials.

### Selection of studies

Two review authors (DGF and RVD) performed an initial screen of titles and abstracts retrieved by the search, and we retrieved the full text of all potentially eligible studies. Two review authors (DGF and RVD) independently examined these studies for compliance with the inclusion criteria and selected studies that met these criteria. Disagreements regarding eligibility were resolved by discussion or by consulting a third review author (JR).

### Inclusion criteria

RCTs were included in the review if the women participating in the trial were of reproductive age (14 years of age or older) diagnosed as having acute PID (symptoms for less than 6 weeks) based on clinical findings, laparoscopy, endometrial biopsy, or detectable *N. gonorrhoeae* or *C. trachomatis* in the upper genital tract. We limited our review to comparison of drugs in current use that are recommended for consideration by the 2015 CDC guidelines for treatment of PID.^2^

### Outcomes

The primary outcomes were clinical cure according to the criteria defined by the treating physician (eg, resolution or improvement of signs and symptoms related to PID) and antibiotic-related adverse events leading to discontinuation of therapy. For secondary outcomes, please see the full Cochrane systematic review.^12^

Where studies included women with various types of pelvic infection, we considered only women with endometritis, salpingitis, parametritis or oophoritis (not related to labour, delivery, cancer or surgery). Where studies reported multiple time points, we included outcomes at between 14 and 28 days after initiation of treatment.

### Data extraction

Data from each study were extracted independently by two of the three review authors (SF, DGF, RVD) using a data extraction form that the review authors designed and pilot-tested. Disagreements were resolved by consensus or by consulting a fourth review author (JR or RFS). If a study had more than two intervention arms, we included or combined only those that met the predefined inclusion criteria. Where studies had multiple publications, we used the main trial report as the reference and derived additional details from secondary papers. We corresponded with study investigators for further data as required.

### Quality of evidence

For each included trial, three review authors (SF, DGF, RVD) independently assessed the risk of bias using the criteria outlined in the Cochrane guidelines.^13^ Disagreements were resolved by discussion or by involving a third review author (JR or RFS).

Two reviewers (SF, RFS) independently assessed and graded the evidence according to the Grading of Recommendations Assessment, Development and Evaluation (GRADE).^15^ Disagreements were resolved by discussion or by involving a third review author (JR). The GRADE tables are available in the full Cochrane review.^12^

### Data synthesis and analysis

Data analyses were performed using Review Manager V.5.3.^16^ A fixed-effect meta-analysis was used for combining data where it was reasonable to assume that trials were estimating the same underlying treatment effect (ie, where trials were examining the same intervention, and the trials’ populations and methods were sufficiently similar). We conducted separate analyses for mild-moderate and severe PID based on the CDC criteria.^2^ If there was clinical heterogeneity sufficient to expect that the underlying treatment effects differed between trials, or if substantial statistical heterogeneity was detected (I^2^=40% or greater), a random-effects meta-analysis was planned to produce an overall summary if a mean treatment effect across trials was considered to be clinically meaningful. For random-effects analyses, the results are presented as the mean treatment effect with 95% CIs, and the estimates of the tau^2^ and I^2^ statistics.

For dichotomous data, the number of events in the control and intervention groups was used to calculate the Mantel-Haenszel risk ratios (RR). For the number needed to treat for an additional beneficial (NNTB) or harmful (NNTH) outcome, the recommendations given by Altman were followed.^17^ When we observed a treatment effect, we reported the NNTH or NNTB with 95% CIs. NNTB and NNTH are presented in the GRADE tables in the full Cochrane review.^12^ When possible, we performed analysis based on intention to treat (ITT). When information for an ITT analysis was not available, we used the results provided by the authors.

We undertook the following sensitivity analysis to investigate whether our conclusions were robust to methodological decisions made by the review authors:

- Risk of bias (restricting analysis to blinded studies at low risk of selection bias).

## Results

**Figure 1:**
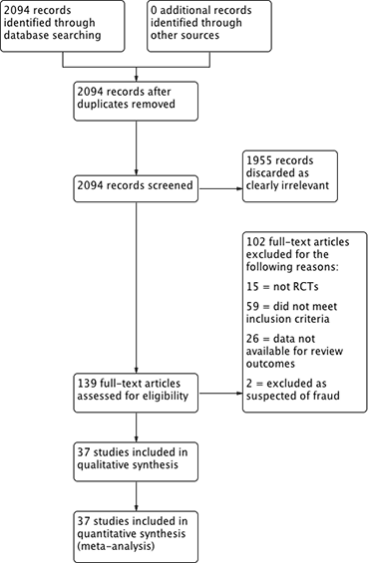
Study flow diagram. RCTs, randomised controlled trials.

We identified 2133 records. After selection process, 37 studies met our inclusion criteria (figure 1).^18–54^

The characteristics of the included RCTs are presented in online supplementary material 1. The 37 trials included 6348 women, with a sample size ranging from 25^18^ to 1156.^19^ Retrieved studies came from a wide range of inpatient and outpatient settings from different continents (North America, South America, Europe, Asia, Oceania and Africa). The trials recruited women aged 14 years and over with a diagnosis of PID according to the CDC criteria (pelvic or lower abdominal pain and one or more of the following clinical criteria: cervical motion tenderness, uterine tenderness or adnexal tenderness).^2^ Studies varied in the degree of disease severity of participants.

10.1136/sextrans-2018-053693.supp1 Supplementary data

### Clinical cure in mild-moderate PID

Two trials compared azithromycin versus doxycycline in mild-moderate PID.^20 21^ There was no clear evidence of a difference between azithromycin and doxycycline regimens (RR 1.18, 95% CI 0.89 to 1.55; 243 women, 2 studies; I^2^=72%; very low-quality evidence). Sensitivity analysis limited to the study at low risk of bias indicated that azithromycin was superior to doxycycline in achieving cure in mild-moderate PID (RR 1.35, 95% CI 1.10 to 1.67; 133 women, 1 study; moderate-quality evidence).^21^

Three studies compared quinolones versus cephalosporins.^22–24^ There was no clear evidence of a difference between the groups (RR 1.04, 95% CI 0.98 to 1.10; 459 women, 3 studies; I^2^=5%; low-quality evidence).

**Figure 2:**
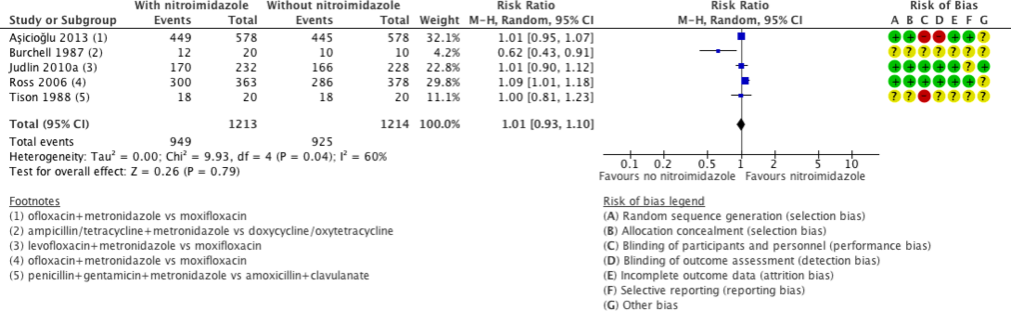
Clinical cure in mild-moderate pelvic inflammatory disease in regimens containing nitroimidazoles versus without nitroimidazoles. (+) low risk of bias, (−) high risk of bias, (?) unclear risk of bias. M-H, Mantel-Haenszel.

Clinical cure in mild-moderate PID was evaluated in five studies comparing nitroimidazoles versus no nitroimidazoles.^19 25–28^ In all the studies the nitroimidazole used was metronidazole. There was no conclusive evidence of a difference in effectiveness between metronidazole versus no use of metronidazole in mild-moderate PID (moderate-quality evidence; figure 2). Sensitivity analysis restricted to the two studies at low risk of bias did not substantially change the main findings (RR 1.06, 95% CI 0.98 to 1.15; 1201 women, 2 studies; I^2^=32%; high-quality evidence).^25 26^

Clindamycin plus aminoglycoside versus quinolone in mild-moderate PID was evaluated in one study,^18^ which showed no difference in effectiveness between the regimens (RR 0.88, 95% CI 0.69 to 1.13; 25 women, 1 study; I^2^=0%; very low-quality evidence).

Two studies compared clindamycin plus aminoglycoside versus cephalosporin in mild-moderate PID.^29 30^ There was no clear evidence of a difference between these regimens in the rates of cure for mild-moderate PID (RR 1.02, 95% CI 0.95 to 1.09; 150 women, 2 studies; I^2^=0%; low-quality evidence).

### Clinical cure in severe PID

One trial compared azithromycin versus doxycycline in severe PID.^31^ There was no clear evidence of a difference in the rates of cure between regimens using azithromycin or doxycycline to treat severe PID (RR 1.00, 95% CI 0.96 to 1.05; 309 women, 1 study; low-quality evidence).

Two studies compared quinolones versus cephalosporins,^32 33^ with no clear evidence of a difference in the rates of cure between regimens (RR 1.06, 95% CI 0.91 to 1.23; 313 women, 2 studies; I^2^=7%; low-quality evidence).

**Figure 3:**
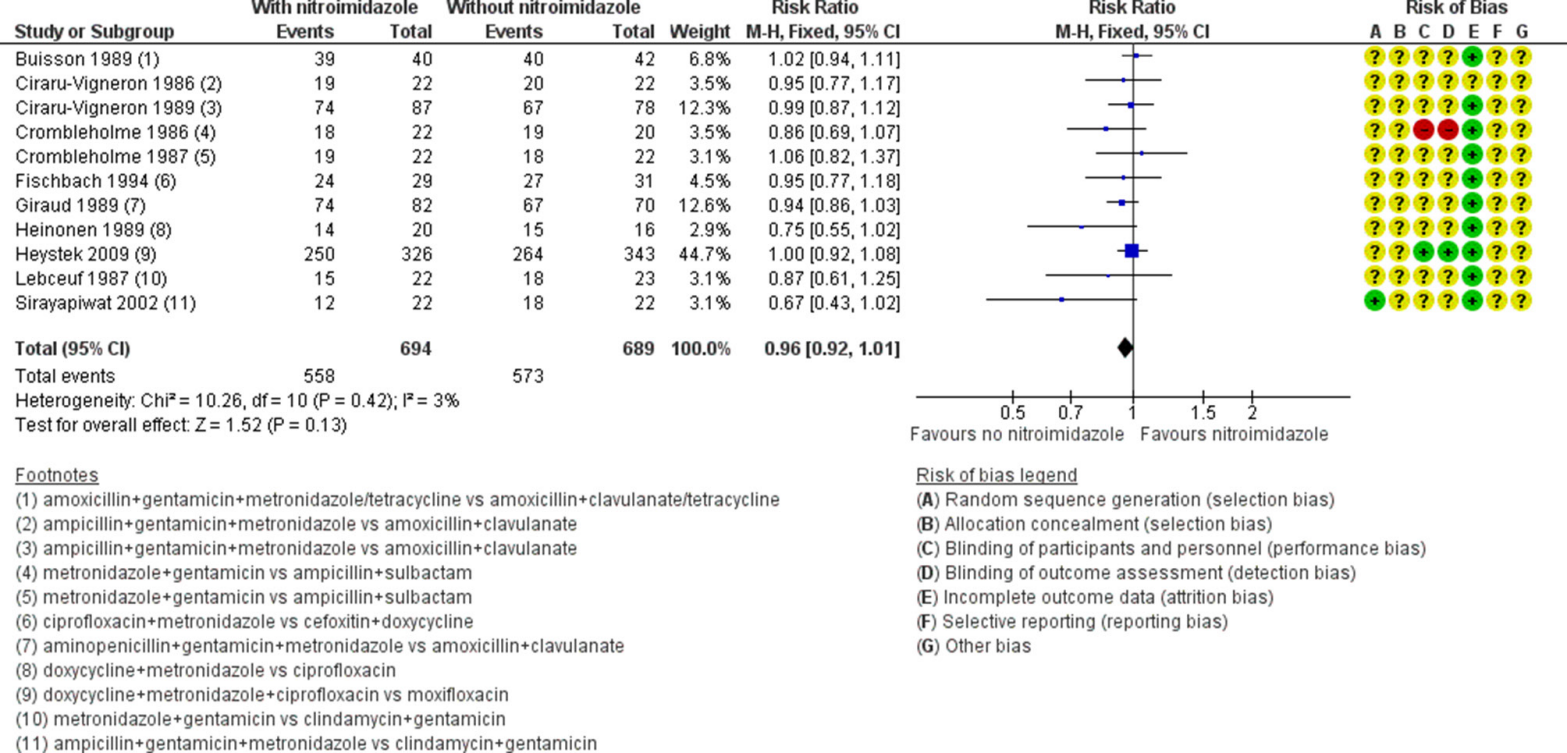
Clinical cure in severe pelvic inflammatory disease in regimens containing nitroimidazoles versus without nitroimidazoles. (+) low risk of bias, (−) high risk of bias, (?) unclear risk of bias. M-H, Mantel-Haenszel.

Eleven studies evaluated nitroimidazole in severe PID, and all studies used metronidazole.^33–43^ The difference in clinical cure rates between women treated with metronidazole and women not treated with it was small and was compatible with no effect (RR 0.96, 95% CI 0.92 to 1.01; 1383 women, 11 trials; I^2^=3%; moderate-quality evidence; figure 3).

Two studies compared clindamycin plus aminoglycoside versus quinolone in severe PID.^44 45^ There was no clear evidence of a difference between these regimens in the rates of cure for severe PID (RR 1.02, 95% CI 0.87 to 1.19; 151 women, 2 studies; I^2^=0%; low-quality evidence).

**Figure 4:**
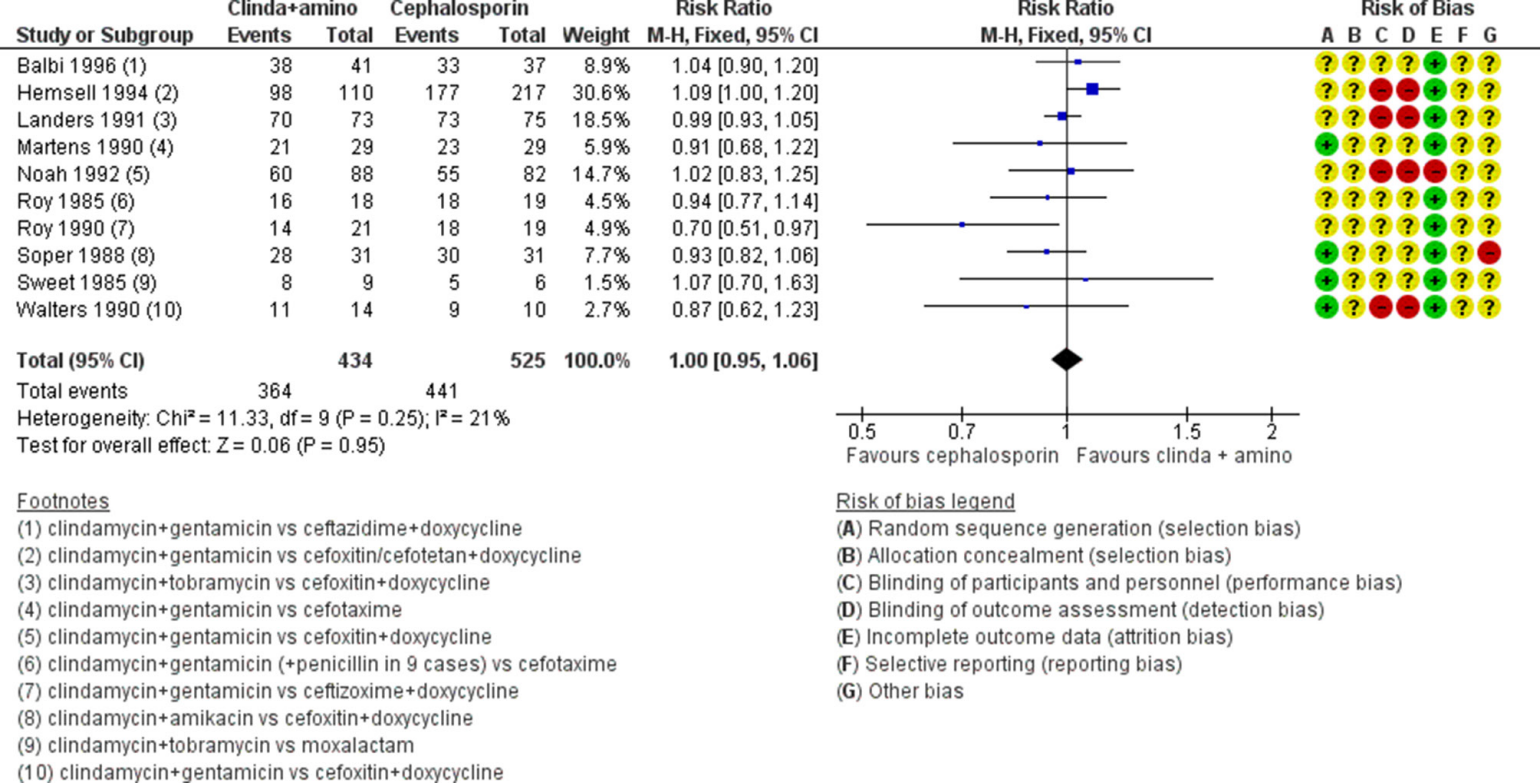
Clinical cure in severe pelvic inflammatory disease in regimens containing clindamycin plus aminoglycoside versus cephalosporin. (+) low risk of bias, (−) high risk of bias, (?) unclear risk of bias. M-H, Mantel-Haenszel.

The use of clindamycin plus aminoglycoside versus cephalosporin in severe PID was evaluated in 10 studies.^29 30 46–53^ There was no clear evidence of a difference between these regimens in the rates of cure of severe PID (moderate-quality evidence; figure 4).

### Adverse events

No clear evidence of a difference between regimens in antibiotic-related adverse events leading to discontinuation of therapy was observed for all comparisons.

### Quality of the evidence

Most of the 37 included studies had unclear or high risk of bias in most domains, and only three were at low risk of bias in most domains.^21 25 26^ The overall quality of the evidence ranged from very low to high, the main limitations being serious risk of bias (due to poor reporting of study methods and lack of blinding), serious inconsistency and serious imprecision. The only high-quality evidence was for the sensitivity analysis regarding the use (or not) of nitroimidazole. There was moderate-quality evidence in the sensitivity analysis regarding the use of azithromycin in mild-moderate cases of PID, in comparisons between the use or not of nitroimidazole for curing mild-moderate or severe PID, and in comparisons between clindamycin plus aminoglycoside versus cephalosporins for curing severe PID.

## Discussion

Thirty-seven trials with 6348 women were included in the review. We found no clear evidence of a difference between any of the regimens studied in terms of effectiveness or safety. Within a sensitivity analysis of cases of mild-moderate PID for the comparison of macrolide (azithromycin) versus tetracycline (doxycycline), we identified a single study at low risk of bias which provided moderate-quality evidence that azithromycin was superior to doxycycline in achieving clinical cure. Some guidelines have recommended the use of nitroimidazoles for PID,^2 11^ but we found no conclusive evidence of a difference in outcomes between the use or not of nitroimidazoles (metronidazole) in successfully treating PID. There was also no clear evidence of a difference in rates of adverse effects between the regimens.

The applicability of the evidence to the target population (women of reproductive age diagnosed with PID) was broad because the included trials were conducted in different clinical settings and implemented varying diagnostic approaches. Additionally, the interventions analysed in the review are currently available and represent the most frequently used treatment regimens in current clinical practice. Given these factors, we consider that the evidence identified applies to a wide range of women with PID varying in disease severity, age, geographical location and diagnostic criteria, which provides external validity.

The trials included in this review cover a period of approximately 30 years and several countries, with 7 out of 37 studies being conducted after the year 2000. The searches for this review were last updated in July 2016 and new evidence may now be available. Considering the availability of additional studies, an update of this review is expected to take place in 2020. Little data are available on temporal variation in bacterial causes of PID because few countries systematically collect this information. Wide variations in the bacterial aetiology of PID may occur in different geographical areas and these may affect the choice of treatment, but few trials were carried out in low-income/middle-income countries. Although we consider that the results of this review are generalisable to a wide range of geographical locations, our conclusions may not be generalisable to low-income/middle-income countries.

An important limitation of this systematic review was the potential for measurement bias introduced by using the investigators’ definitions of cure. This approach was necessary because of the wide variation in methods used and lack of a widely accepted objective outcome measure. The short-term follow-up in most studies prevented the identification of long-term sequelae. In addition, the inaccuracy of clinical diagnosis for PID and the wide variety of assessment criteria used for clinical cure may have reduced the power of the analysis to detect a clinically relevant effect. Some studies identified PID and endometritis separately, but these were pooled for our analysis. Data were lacking for several of our secondary outcomes. None of the included studies reported data on fertility or laparoscopic evidence of PID resolution, and data were very scant on length of hospital stay.

Microbiological clearance of *C. trachomatis* and *N. gonorrhoeae* and clinical resolution of symptoms are the usual outcomes used in clinical practice and the current review reflects this.^12^ The utility of other biological markers (eg, mediators of inflammation, ‘new’ bacteria) is being explored, but their use as outcome measures is not yet established even in a research setting.^55 56^ Bacterial sequencing and 16S ribosomal RNA are also experimental, and their role in the diagnosis, or as prognostic markers, remains uncertain.

Increasing resistance in *N. gonorrhoeae* has implications for the treatment of gonococcal PID and strongly suggests that only parenteral third-generation cephalosporins should be used in this situation, but this accounts for a very small proportion of PID overall. Studies have demonstrated that women with PID are often coinfected with other micro-organisms such as *Mycoplasma genitalium*, *Trichomonas vaginalis*, *C. trachomatis* and anaerobes. There is increasing recognition that *M. genitalium* is an important cause of PID, although only in a minority of women. However, few published studies (and no RCTs) have addressed this.^11 57^ The utility of azithromycin as empirical treatment for *M. genitalium* is limited by increasing rates of resistance, and treatment with moxifloxacin is currently recommended for this group of patients.^58 59^ *T. vaginalis* has been associated with endometritis, but its role in PID remains uncertain.^60^ Bacterial vaginosis is not commonly associated with PID in prospective studies, although subgroups of women with bacterial vaginosis may be at higher risk, especially if coinfected with *N. gonorrhoeae* or *C. trachomatis*.^61^

Anaerobic bacteria are commonly identified in the fallopian tubes of women with PID,^62^ and most treatment guidelines include metronidazole to provide adequate microbiological cover. On meta-analysis we found that the addition of a nitroimidazole (metronidazole) did not improve short-term clinical outcomes of either mild-to-moderate or severe PID, which suggests that the other components of treatment regimens may be adequate. This is potentially important since nitroimidazoles commonly cause gastrointestinal side effects and may limit adherence to therapy. Nevertheless, some studies used antimicrobials with anaerobic cover, such as amoxicillin+clavulanate.^28^ The only study, in this systematic review, that did not use any antibiotic with anaerobic coverage found that women who did not receive nitroimidazole were more likely to experience clinical cure than those who received nitroimidazole.^28^ Of note, the study conducted by Burchell *et al* 28 had a small sample size and lacked information to assess risk of bias. We were not able to assess long-term outcomes such as infertility or chronic pelvic pain, and it remains uncertain whether the use of nitroimidazoles affects the risk of these sequelae.

One previous meta-analysis, published in 1993, formed the basis for the CDC guidelines.^63^ The authors reported pooled clinical cure rates ranging from 75% to 94%, which is similar to our updated review with overall rate of cure of 81%. The uncertainty in using nitroimidazoles when treating PID is reflected in the current guidelines. The 2015 US CDC PID guideline^2^ advises that metronidazole be considered to provide additional anaerobic cover but does not mandate its use. The 2017 European IUSTI PID guideline^11^ and the 2018 BASHH PID guideline^64^ recommend the use of metronidazole but advise that it can be discontinued in those with mild to moderate symptoms if they develop drug-related side effects. Our analysis does not support the routine use of metronidazole in the treatment of women with mild to moderate PID and can be used to inform future guideline revisions.

### Differences between the Cochrane review and the current review

The current review is an abridged version of a Cochrane systematic review and presents the main findings and an enhanced discussion section.^12^ The secondary outcomes presented in the Cochrane review were microbiological clearance of *C. trachomatis,* microbiological clearance of *N. gonorrhoeae*, laparoscopic evidence of resolution of PID based on physician opinion, length of stay (for inpatient care) and rate of fertility. The results were similar for studies that reported microbiological clearance of *C. trachomatis* or *N. gonorrhoeae*, with clearance occurring in over 90% of women irrespective of regimen used. Length of stay varied across the studies, ranging from 3 days to 18 days. No data were found for laparoscopic evidence of resolution of PID and for rate of fertility.

## Conclusion

We found no evidence that one regimen is more effective or safer than any other for the treatment of PID, and there is no clear evidence that the addition of nitroimidazoles is beneficial. Adherence to clinical treatment for PID is an important issue that should be considered when choosing a treatment regimen. Moderate-quality evidence from a single study at low risk of bias suggests that a macrolide (azithromycin) may be more effective than a tetracycline (doxycycline) for curing mild-moderate PID. There remains a need for high-quality RCTs to assess treatments for women with PID, particularly comparing regimens with or without the addition of nitroimidazoles and comparing azithromycin (eg, 1 g once a week for 2 weeks) with doxycycline. The lack of a consistent outcome measure to assess response to therapy is a major limitation, and there is a clear need for core outcome measures to be developed.

**Key messages**

- There is no clear evidence that any one of the currently recommended treatment regimens for pelvic inflammatory disease (PID) is superior to another.
- There is no evidence of improved efficacy when metronidazole is included within a treatment regimen, although this is currently recommended in some guidelines.
- There is a need for better understanding of the role of azithromycin in the treatment of PID, given uncertainty about its efficacy compared with doxycycline and concerns about inducing antimicrobial resistance.
