# Supplementary material for: Antibiotic therapy for pelvic inflammatory disease: an abridged version of a Cochrane systematic review and meta-analysis of randomised controlled trials
Source: Sex Transm Infect. 2018 Oct 19;95(1):21–7. doi: 10.1136/sextrans-2018-053693 (PMC6580736; doi:10.1136/sextrans-2018-053693)
Supplement: Supplementary data [file sextrans-2018-053693supp001.pdf]

Supplementary material 1. Characteristics of included randomized controlled trials

First author (year)	Participants and setting	Interventions	Outcomes
Apuzzio (1989) [18]	Number of women randomized: 25 for PID<br>Number of women analyzed: group A: 10; group B: 15<br>Age (years): group A: 23.2; group B: 23.2<br>Number of centres (country): 1 (US)	Group A: ciprofloxacin 300 mg IV every 12 h; treatment continued for 3-5 days until the woman was asymptomatic for 24 h. Upon discharge from hospital, women received oral antibiotics to complete 10-14 days of ciprofloxacin 750 mg PO twice daily<br><br>Group B: clindamycin 900 mg IV every 8 h + gentamicin 1.5 mg/kg IV every 8 h. Upon discharge from the hospital, the women received oral antibiotics to complete 10-14 days of clindamycin 450 mg PO every 6 h	Primary outcome: treatment success, defined as women asymptomatic for 24h.
Arredondo (1997) [24]	Number of women randomized: 69 in each group<br>Number of women analyzed: 69 in each group<br>Age (mean) (years): group A: 28.9; group B: 30.7<br>Number of centres (country): 6; Chile (1 centre), Peru (2 centres), Colombia (2 centres), and Mexico (1 centre)	Group A: clindamycin 300 mg (2 capsules 3 times daily) + ciprofloxacin (250 mg, 1 tablet twice daily) for 14 days and placebo IM (for an equivalent of 1 dose of ceftriaxone)<br><br>Group B: ceftriaxone 250 mg IM (as a single dose) + doxycycline 100 mg (1 capsule twice daily) and placebo (2 capsules 3 times daily for equivalent doses of clindamycin) for 14 days	Primary outcome: clinical cure defined by the absence of, or minimal, pelvic tenderness, temperature <37.5 °C, and a WBC count of 10,000/mm3, if a minimum of 4 days of treatment had been completed. Clinical improvement defined as resolution of 2 of these 3 symptoms.<br>Secondary outcomes: failure when 1 of the following circumstances was noted after at least 48 h of protocol therapy: signs and symptoms remained unchanged or worsened (during the first 72 h of therapy).<br>Microbiological cure defined as eradication of *Neisseria gonorrhoeae* or *Chlamydia trachomatis* (or both) from clinically cured women. Failure defined as persistence of 1 or both of these 2 organisms or, in the case of clinical improvement or failure, the presence of endocervical pathogens. Superinfection defined as the isolation of ≥ 1 new pathogens.<br>Adverse effects leading to discontinuation of treatment.
Aicioglu (2013) [19]	Number of women randomized: 1156<br>Number of women analyzed: group A: 578; group B: 578	Group A: moxifloxacin 400 mg once daily for 14 days<br><br>Group B: ofloxacin 400 mg twice daily + metronidazole 500 mg PO twice daily	Primary outcome: clinical cure, defined as a ≥ 60% reduction in the total pain score at day 21 compared with baseline and the absence of pelvic discomfort and tenderness,

	Age (mean ± SD) (years): group A: 30.3 ± 3.7; group B: 29.3 ± 3.5<br>Number of centres (country): 4 (Turkey)		temperature < 37.8 °C, and WBC < 10,000/mm3 on day 21.<br>Secondary outcomes: microbiological cure, adverse effects.
Balbi (1996) [53]	Number of women randomized: 78<br>Number of women analyzed: 76; group A: 40; group B: 36<br>Age (mean ± SD) (years): group A: 25.3 ± 7.7.4; group B: 29.4 ± 7.8<br>Number of centres (country): 1 (Italy)	Group A: gentamicin, 2 mg/kg IV (attack dose), followed by 1.5 mg/kg IV every 8 h + clindamycin 900 mg IV every 8 h for 4 days, followed by clindamycin 450 mg PO every 6 h for a total of 14 days of treatment<br><br>Group B: ceftazidime 1 g IV every 8 h + doxycycline 100 mg PO every 12 h for 4 days, followed by doxycycline 100 mg PO every 12 h for a total of 14 days of treatment	Primary outcome: clinical recovery, defined as: body temperature < 37 °C per 48 h, disappearance of pelvic pain, no increase of eventual adnexal mass after 7 days of the end of treatment<br>Secondary outcome: follow-up performed 30 days after treatment finished; endocervical culture for *Neisseria gonorrhoeae* and *Chlamydia trachomatis* and endometrial culture for *Chlamydia trachomatis* performed in all positive cases at admission.
Bevan (2003) [31]	Number of women randomized: 310<br>Number of women analyzed: 309; group A: 106; group B: 107; group C: 96<br>Age (mean (range)) (years): group A: 28.4 (18-54); group B: 27.6 (18-46); group C: 27.6 (17-54)<br>Number of centres (country): multiple, but the authors did not specify how many (UK and others that were not stated)	Group A: azithromycin 500 mg IV single dose, days 2-7: 250 mg PO once daily or days 1-2: azithromycin 500 mg IV once daily; days 3-7: 250 mg PO once daily<br><br>Group B: as group A + day 1: metronidazole 500 mg IV 3 times daily or 400 mg PO 3 times daily, days 2-12: metronidazole 400mg PO3 times daily or days 1-2: azithromycin 500 mg IV once daily; days 3-7: 250 mg PO once daily plus day 1-2: metronidazole 500 mg IV 3 times daily, or 500 mg PO 3 times daily. Days 3-12: metronidazole 500 mg PO 3 times daily or days 1-21: metronidazole 500 mg PO 3 times daily<br><br>Group C: day 1: metronidazole 500 mg IV 3 times daily, or metronidazole 400 mg PO 3 times daily; day 2-12: metronidazole 400 mg PO 3 times daily + days 1-14: cefoxitin 2 g IV or IM4 times daily + day 1: probenecid 1 g PO single dose or day 1-21: doxycycline 100 mg PO twice daily + day 1-5: amoxicillin/clavulanate 1 g IV + times daily; day 6-21 amoxicillin/clavulanate 500 mg PO 3 times daily	Primary outcome: clinical response to treatment: cure, resolution of all baseline signs and symptoms; improvement, lessening of the baseline signs and symptoms or absence of ≥ 1, but not all, of the baseline findings; or failure, no improvement or deterioration of baseline condition. Successful clinical outcome defined as cure or improvement. Assessment on day 15 (9-26 inclusive) and at follow-up (day 35-44).<br>Microbiological outcome: eradication, absence of the baseline isolate(s); persistence, presence of baseline isolate(s); or superinfection, presence of a micro-organism different from that found at baseline.
Buisson (1989) [38]	Number of women randomized: 82<br>Number of women analyzed: group A: 42; group B: 40	Group A: amoxicillin-clavulanic acid 1 g IV every 8 h for at least 48 h, then 1.5-2 g PO twice daily. Mean length of treatment 19 days, never less than 14 days	Primary outcomes: clinical cure; defined as absence of fever, pain, and previously observed adnexal masses at 5-8 weeks’

	Age (mean (range)) (years): group A: 27.7 (18-46); group B: 28.7 (15-49)<br>Number of centres (country): 8 (France)	Group B: amoxicillin 3-4 g IV per 24 h, mean 4 days, then 1.5-2 g PO daily. Mean length of treatment 17 days + aminoside (chosen by researcher's preference) 3-5 mg/kg IM per 24 h 2 or 3 times daily depending of the aminoside, mean length of treatment 7 days + metronidazole 1 or 1.5 g IV or suppository daily.<br><br>For each case, a secondary prescription for a tetracycline 200 mg per 24 h was given, either immediately if results of laparoscopy or other investigations justified it, or later on positive *Chlamydia trachomatis* serology. Length of this treatment 3-4 weeks as decided by the researcher	follow-up; adverse events leading to discontinuation of treatment
Burchell (1987) [28]	Number of women randomized: 30 women, 10 in each group<br>Number of women analyzed: 30 women, 10 in each group<br>Age (years): not stated.<br>Number of centres (country): not stated (South Africa)	Group A: doxycycline infusion 200 mg in 200 ml 5% dextrose over 2 h + doxycycline 100 mg after 24 h. The course was completed with oxytetracycline 250 mg every 6 h for 14 days<br><br>Group B: ampicillin + metronidazole every 6 h 1 g IV and then 500 mg PO every 6 h for 14 days<br><br>Group C: tetracycline + metronidazole with 3 × 1 g suppositories every 8 h and then 400 mg PO every 8 h for 14 days	Primary outcome: clinical cure
Ciraru-Vigneron (1986) [34]	Number of women randomized: 44; 22 in each group<br>Number of women analyzed: 44; 22 in each group<br>Number of centres (country): 1 (France)<br>Age (years): not stated	Group A: amoxicillin-clavulanic acid while in hospital, 4 g per 24 h, first by IV, then PO once symptoms improved. At discharge, amoxicillin-clavulanic acid PO if *Chlamydia trachomatis* serology was negative; if positive for *Chlamydia trachomatis*, doxycycline prescribed for 3 weeks (dose not stated)<br><br>Group B: ampicillin 6 g per 24 h IV+ gentamicin 160mg per 24 h IM+metronidazole 1.5 g per 24 h IV; when switched to oral administration, ampicillin replaced by amoxicillin 3 g per 24 h + metronidazole 1.5 g per 24 h PO and gentamicin 160 mg per 24 h IM for minimum of 7 days. At discharge, amoxicillin-clavulanic acid PO if *Chlamydia trachomatis* serology negative; if positive for *Chlamydia trachomatis*, doxycycline for 3 weeks (dose not stated)	Primary outcomes: clinical cure, defined by no fever, reduction of pain, and normal WBC count at discharge and in 30 days; adverse events leading to discontinuation of treatment<br>Secondary outcomes: microbial cure of *Neisseria gonorrhoeae* and *Chlamydia trachomatis*; length of hospital stay
Ciraru-Vigneron (1989) [35]	Number of women randomized: 165 women; group A: 78; group B: 87	Group A: amoxicillin 1 g IV + clavulanic acid 200 mg (Augmentin 1.2 g), 3 or 4 times daily until clinical and laboratory findings	Primary outcomes: excellent response defined as resolution of physical findings with continued improvements in laboratory values;

	Number of women analyzed: 152 women; group A: 70; group B: 82<br>Number of centres (country): 8 (France)<br>Age (mean) (years): group A: 26.9; group B: 25.1	improved, after which 4-6 tablets containing amoxicillin 500 mg + clavulanic acid 125 mg (Augmentin 625 mg) per tablet<br><br>Group B: amoxicillin or ampicillin 3-4 g daily, with an aminoglycoside (gentamicin 160 mg, dibekacin 150 mg, or tobramycin 150 mg) + metronidazole 1.5 g daily parenterally. Subsequent conversion was to oral combination of amoxicillin or ampicillin 2-3 g + metronidazole 1-1.5 g daily	favourable response equate with a favourable course, allowing the persistence of ≥ 1 clinical signs or abnormal laboratory values (or both); failure defined as absence of therapeutic efficacy or of a favourable course after at least 6 days of therapy that a change in management, either surgery or an alternative antibiotic treatment was warranted
Crombleholme (1986) [36]	Number of women randomized: 39; group A: 20; group B: 19<br>Number of women analyzed: 39; group A: 20; group B: 19<br>Age (years): not stated.<br>Number of centres (country): not stated (not stated)	Group A: sulbactam 1 g + ampicillin 2 g IV every 6 h<br><br>Group B: metronidazole 15 mg/kg loading followed by 7.5 mg/kg IV every 6 h and gentamicin 1.5 mg/kg IV every 8 h<br><br>Antibiotics in both groups continued for minimum of 5 days	No relevant outcomes reported for our analysis because the outcomes were not separately reported for the different diagnoses within the study groups. Authors stated clinical cure occurred in 19/20 women in group A and 16/19 women in group B
Crombleholme (1987) [37]	Number of women randomized: 44; 22 in each group<br>Number of women analyzed: 42; 21 in each group<br>Age (mean ± SD) (years): group A: 27.7 ± 6.9; group B: 29.0 ± 12.1<br>Number of centres (country): 1 (US)	Group A: ampicillin 2 g + sulbactam 1 g IV every 6 h.<br><br>Group B: metronidazole 15 mg/kg IV every 6 h + gentamicin 1.5 mg/kg IV every 8 h<br><br>Therapy continued until women became afebrile and were without clinical signs of infection for 48 h or until clinical judgement dictated cessation of therapy. Range of treatment 3-11 days.	Primary outcome: clinical cure defined as absence of fever, without clinical signs of infections for 48 h or until clinical judgement
Crombleholme (1989) [44]	Number of women randomized: 80; 40 in each group<br>Number of women analyzed: 80; 40 in each group<br>Age (mean ± SD) (years): group A: 25.7 ± 4.8; group B: 25.3 ± 6.2<br>Number of centres (country): 1 (US)	Group A: ciprofloxacin 300 mg IV every 12 h for 2-5 days, with ≥ 2 days of parenteral therapy and 48 h without fever before switching to ciprofloxacin 750 mg PO every 12 h, to complete a 14-day course<br><br>Group B: clindamycin 600 mg IV every 6 h + 1 mg/kg IV every 8 h after ≥ 4 days of parenteral gentamicin and 48 h without fever before switching to clindamycin 300 mg PO every 6 h, to complete a 14-day course	Primary outcome: clinical cure at 3 days: improvement in clinical signs and symptoms<br>Secondary outcomes: microbial cure of *Chlamydia trachomatis* and *Neisseria gonorrhoeae*
European Study Group (1992) [50]	Number of women randomized: 170 women; group A: 88; group B: 82<br>Number of women analyzed: 170 women; group A: 88; group B: 82	Group A: clindamycin 900 mg IV every 8 h + gentamicin 2 mg/kg IV, followed by 1.5 mg/kg IV every 8 h for minimum of 4 days. At the end of the period of IV therapy, clindamycin 450 mg PO every 6 h was given to complete 14 days of treatment	Primary outcomes: clinical failure, minimum of 48 h of protocol therapy and characterized by signs and symptoms as unchanged or worsened during the first 48-72 h of

	Age (mean) (years): whole study group: 28; age per group not stated.<br>Number of centres (country): 10 (9 in Europe and 1 Africa)	Group B: cefoxitin 2 g IV every 6 h + doxycycline 100 mg IV every 12 h were given for at least 4 days. At the end of IV therapy, doxycycline 100 mg PO was given every 12 h to complete a total of 14 days of treatment	treatment, or worsening later, failure to improve further; need of additional antibiotics or need for surgery considered as failure; adverse events leading to discontinuation of therapy<br>Secondary outcomes: microbial cure of *Chlamydia trachomatis* and *Neisseria gonorrhoeae*
Fischbach (1994) [33]	Number of women randomized: 60<br>Number of women analyzed: 57; group A: 26; group B: 31<br>Age (average (range)) (years): group A: 27 (30 ± 10); group B: 22 (26 ± 7)<br>Number of centres (country): not stated (Germany)	Group A: ciprofloxacin 2 × 0.2 g IV daily + metronidazole 3 × 0.5 g IV daily<br><br>Group B: cefoxitin 3 × 2 g IV daily + doxycycline 2 × 0.1 g IV daily<br><br>After 2-5 days of treatment, ciprofloxacin, metronidazole, and doxycycline given PO. Both groups treated for 7-14 days	Primary outcome: clinical cure defined as subjective lack of symptoms, improvement upon gynaecological examination, normal leukocytes count, declining ESR, no fever, and elimination of bacteria
Giraud (1989) [39]	Number of women randomized: 152<br>Number of women analyzed: group A: 70; group B: 82<br>Age (mean) (years): group A: 26.9; group B: 25.1<br>Number of centres (country): unclear (France)	Group A: amoxicillin-clavulanic acid; while in hospital, 3 or 4 g per 24 h, by IV or perfusion, then 2 or 3 g per 24 h PO<br><br>Group B: parenteral: ampicillin 3 or 4 g IV per 24 h, OR amoxicillin 3 g IV per 24 h + gentamicin 160 mg IM per 24 h OR dibekacin 150 g IM per 24 h + metronidazole 1.5 g IV per 24 h. Oral administration: ampicillin 3 g per 24 h OR amoxicillin 2 g/24 h + metronidazole 1.5 or 2 g per 24 h<br><br>Additional treatments were limited, but could include: surgical or laparoscopic drainage of pus, local antibiotic for treatment of trichomonacides, non-steroidal anti-inflammatory drugs, corticoids	Primary outcomes: clinical cure at 10th day; clinical progress and improvement of biological parameters<br>Secondary outcome: length of hospital stay
Heinonen (1989) [40]	Number of women randomized: 40<br>Number of women analyzed: group A: 16; group B: 20<br>Age (mean ± SD (range)) (years): group A: 29 ± 8 (18-43); group B: 29 ± 1 (16-50)<br>Number of centres (country): 1 (Finland)	Group A: ciprofloxacin 200 mg IV every 12 h for 2 days followed by 750 mg PO every 12 h to complete 14-day course<br><br>Group B: doxycycline 100 mg IV every 12 h + metronidazole 500 mg IV every 8 h for the first 2 days, followed by doxycycline 150 mg PO every 24 h + metronidazole 400 mg PO every 8 h to complete a 14-day course	Primary outcomes: clinical response based on a scale from 0 (absent or normal) to 3+ (severe) assessed on days 3, 6, 14, and 21 after the antimicrobial treatment was started. Failure defined as the presence of ≥ 1 of following criteria: no improvement in the clinical severity score at day 3 after the microbial treatment was started; CRP > 20 mg/L or a decline < 50% in the initial CRP level

			at day 6; positive cervical culture of *Neisseria gonorrhoeae* or *Chlamydia trachomatis* at days 14 or 21; or the need for additional antimicrobial agents or surgical intervention<br>Secondary outcomes: adverse reactions and effects
Hemsell (1994) [52]	Number of women randomized: 344<br>Number of women analyzed: group A: 109; group B: 110; group C: 108<br>Age (mean ± SD) (years): success group: 24.7 ± 4.9; failure group: 26.2 ± 5.7<br>Number of centres (country): 6 (US)	Group A: cefoxitin 2 g IV every 6 h + doxycycline 100 mg every 12 h; followed by doxycycline 100 mg PO twice daily for a total of 10-14 days<br><br>Group B: clindamycin 900 mg IV + gentamicin 1.5 mg/kg every 8 h after an initial gentamicin loading dose calculated at 2 mg/kg followed by clindamycin 450 mg PO 4 times daily for a total of 10-14 days<br><br>Group C: cefotetan 2 g IV + doxycycline 100 mg every 12 h, followed by doxycycline 100 mg PO twice daily for a total of 10-14 days	Primary outcome: clinical cure defined as reduction of the severity score by ≥ 70%, with a normal temperature and leukocyte count
Heystek (2009) [41]	Number of women randomized: 686<br>Number of women analyzed: 669; group A: 343; group B: 326<br>Age (mean ± SD) (years): group A: 29.0 ± 7.3; group B: 28.2 ± 7.2<br>Number of centres (country): 43 (14 countries)	Group A: moxifloxacin 400 mg PO once daily for 14 days<br><br>Group B: doxycycline 100 mg PO twice daily + metronidazole 400 mg PO 3 times daily for 14 days + ciprofloxacin 500 mg PO once daily for 14 days	Primary outcome (modified ITT): clinical cure at 2-14 days' post treatment: clinical success defined as cure (severity score reduced by ≥70%plus normal temperature and leukocyte count) or improvement (severity score reduced <70% but >30% plus normal temperature and leukocyte count). Therapy considered to have failed if symptoms and signs of infection persisted or worsened, as shown by persistent fever, leukocytosis, a reduction in severity score of ≤30%, or a combination of these. Clinical efficacy was 'unevaluable' when a woman could not be assessed<br>Secondary outcome (PP): microbiological clearance of *Chlamydia trachomatis*, microbiological clearance of *Neisseria gonorrhoeae*

Hoyme (1993) [54]	Number of women randomized: 33<br>Number of women analyzed: 33; group A: 15; group B: 18<br>Age (years): not stated<br>Number of centres (country): 1 (Germany)	Group 1: ofloxacin 2 × 200 mg + metronidazole 2 × 500 mg, first IV and then PO for 10 days in total<br><br>Group 2: gentamicin 3 × 80 mg + clindamycin 4 × 600 mg (initially 1200 mg IV) for 10 days in total	Primary outcome: clinical cure, no raw data reported for outcomes
Judlin (2010) [25]	Number of women randomized: 460<br>Number of women analyzed: ITT: group A: 228; group B: 232; PP: group A: 194; group B: 190<br>Age (mean ± SD) (years): group A: 35.2 ± 8.4; group B: 35.4 ± 8.7<br>Number of centres (country): 7 (China, Indonesia, South Korea,The Philippines, Pakistan, Thailand, Taiwan)	Group A: moxifloxacin 400 mg PO once daily for 14 days<br><br>Group B: levofloxacin 500 mg (2 x 250 mg tablets) PO once daily + metronidazole 500 mg (1 tablet) PO twice daily for 14 days	Efficacy: primary efficacy variable was clinical response at test-of-cure in the PP population: ’Clinical success’ defined as women with clinical cure at test-of-cure; failures were women with failure at the ’during therapy’ visit or improvement or failure at test-of cure. ’Clinical cure’ defined as reduction in tenderness score (McCormack scale) of >70%, apyrexia (rectal/tympanic/oral temperature < 38.0 °C or axillary temperature <37.5 °C) and WBC < 10,500/mm^3. ’Clinical improvement’ defined as reduction in tenderness score of 30-70%, apyrexia (rectal/tympanic/oral temperature < 38.0 °C or axillary temperature < 37.5°C) and WBC < 10,500/mm^3. ’Clinical failure’ defined as reduction in tenderness score of < 30% or elevated temperature (rectal/tympanic/oral temperature ≥ 38.0 °C or axillary temperature < 37.5 °C) or WBC ≥ 10,500/mm^3, or a combination of these. Secondary efficacy variables: Clinical response during therapy (PP population) classified as ‘clinical improvement’, defined as reduction in tenderness score of > 30% with improvement in temperature, or ’clinical failure’, defined as persistence or worsening of symptoms and signs of infection, as evidenced by a reduction in tenderness score of > 30% or no improvement in temperature (or both)

Landers (1991) [51]	Number of women randomized: 162<br>Number of women analyzed: 148, group A: 75; group B: 73<br>Age (mean ± SD) (years): total: 23.5 ± 6.1; group A: 23.3 ± 5.3; group B: 23.8 ± 6.0<br>Number of centres (country): 2 (US)	Group A: cefoxitin 2 g IV every 6 h + doxycycline 100 mg IV every 12 h for minimum 4 days and at least 48 h after disappearance of fever. Women without fever on admission treated for minimum of 4 days in the hospital for at least 24 h beyond the adequate relief of pain and tenderness to a normal lifestyle without surgical intervention. After discharge from the hospital, doxycycline 100 mg PO twice daily was continued to complete a total of 14 days of treatment<br><br>Group B: clindamycin 600 mg IV every 6 h + tobramycin 2 mg/kg IV for 1 dose, followed by 1.5 mg/kg IV every 8 h for minimum of 4 days and for at least 48 h after disappearance of fever. Women without fever on admission treated for minimum of 4 days in hospital for at least 24 h beyond adequate relief of pain and tenderness to a normal lifestyle without surgical intervention. After discharge from hospital, clindamycin 450 mg PO 4 times daily continued to complete a total of 14 days of treatment	Primary outcome (ITT): clinical response for a satisfactory initial clinical response defined as an improvement of admitting signs and symptoms, included abdominal-pelvic pain, fever, and pelvic tenderness. Follow-up evaluation performed at hospital discharge and at 2-6 weeks after initial enrolment<br>Secondary outcomes (PP): microbiological clearance of *Chlamydia trachomatis* and reduction in tenderness score
Leboeuf (1987) [42]	Number of women randomized: 45; group A: 23; group B: 22<br>Number of women analyzed: 39; group A: 21; group B: 18<br>Age (mean ± SD) (years): group A: 27.9 ± 5.2; group B: 29.1 ± 9.5<br>Number of centres (country): 2 (France)	Group A: clindamycin 900mg IV every 8 h diluted in 150mL (minimum volume) saline slow perfusion (30-60 minutes) + gentamicin 1 mg/kg IM every 8 h (with minimum dose prescribed of 60 mg every 8 h, according to bodyweight). Treatment given for minimum of 5 days in hospital. Clindamycin perfusion not stopped until 48 consecutive h with temperature below 37.5 °C; at that point treatment could be PO. Maximum length of treatment at the discretion of the therapist. If also treated with a tetracycline, this was not prescribed < 48 h after the end of the treatment protocol (either clindamycin + gentamicin or metronidazole + gentamicin)<br><br>Group B: metronidazole 500 mg every 8 h in slow IV perfusion (30-60 minutes) + gentamicin 1 mg/kg IM every 8 h (with minimum dose prescribed of 60 mg every 8 h, according to bodyweight). Treatment given for 6 weeks	Primary outcome: clinical cure: absence of infection in the days following cessation of treatment according to clinical observations, microbe eradicated during or after treatment<br>Secondary outcome: length of hospital stay
Malhotra (2003) [20]	Number of women randomized: 165<br>Number of women analyzed: 153 women; group A: 52; group B: 48; group C: 53	Group A: ciprofloxacin 500mg + tinidazole 600mg twice daily for 7 days<br><br>Group B: fluconazole 150 mg (1 tablet) + azithromycin 1 g (1 tablet) + secnidazole 2 g (2 tablets). Advised to take azithromycin	Primary outcome: clinical cure defined as at least 70% reduction in severity score, no more than mild abdominal pain, and no recurrence of symptoms or signs of PID within 4 weeks of

	Age (mean ±SD) (years): group A: 25.6 ± 3.9; group B: 25.8 ± 3.2; group C: 25.5 ± 4.9<br>Number of centres (country): 1 (India)	on empty stomach in the morning, secnidazole with or after food and fluconazole in the evening<br><br>Group C: doxycycline 100 mg twice daily + metronidazole 200 mg 3 times daily for 1 week. The 2 drugs were available in the hospital pharmacy free of cost	therapy. Treatment failure defined as < 20% decrease in tenderness score
Martens (1990) [49]	Number of women randomized: 99<br>Number of women analyzed: 94<br>Age (mean ± SD) (years): group A: 26 ± 7; group B: 25 ± 7; group C: 26 ± 7<br>Number of centres (country): not stated (not stated)	Group A: cefoxitin 2 g every 6 h for minimum of 4 days and continued until the woman was apyrexial with improvement of symptoms for at least 48 h<br><br>Group B: cefotaxime 2 g every 8 h for minimum of 4 days and continued until the woman was apyrexial with improvement of symptoms for at least 48 h<br><br>Group C: clindamycin 900 mg every 8 h + gentamicin at initial loading dose of 120 mg, followed by maintenance doses of 80 mg every 8 h. Subsequent maintenance doses determined by evaluating trough and peak serum aminoglycoside concentrations. Antibiotics given for minimum of 4 days, and continued until the woman was apyrexial with improvement of symptoms for at least 48 h	Primary outcome: clinical failure: evaluated on a daily basis. Women who had not demonstrated signs of improvement after 48-72 h of antibiotic therapy were considered an unsuccessful result
Martens (1993) [23]	Number of women randomized: total: 295; group A: 150; group B: 145<br>Number of women analyzed: total: 249; group A: 128; group B: 121<br>Age (mean ± SD) (years): group A: 25.9 ± 5.8; group B: 26.0 ± 6.8<br>Number of centres (country): 16 (US)	Group A: ofloxacin 400 mg PO every 12 h for 10 days<br><br>Group B: cefoxitin 2 g IM + probenecid 1 g PO, followed by doxycycline 100 mg PO every 12 h for 10 days	Primary outcomes: clinical cure: complete resolution of tenderness; clinical improvement: partial resolution of tenderness without the need for additional antibiotic therapy<br>Secondary outcomes: microbial cure of *Chlamydia trachomatis*, microbial cure of *Neisseria gonorrhoeae*
Okada (1988) [32]	Number of women randomized: total: 253; group A: 124; group B: 129<br>Number of women analyzed: total: 209; group A: 104; group B: 105<br>Age (years): only presented as frequencies within age groups<br>Number of centres (country): 55 (Japan)	Group A: ciprofloxacin 200 mg PO + dummy cefroxadine placebo, 3 times daily for 7 consecutive days<br><br>Group B: cefroxadine 250 mg PO + dummy ciprofloxacin placebo, 3 times daily for 7 consecutive days	Primary outcomes: clinical cure at 7th day of treatment: excellent: clinical marked improvement and clearance of bacteria; good: clear clinical improvement; poor: no clear clinical improvement; adverse events<br>Secondary outcome: local tenderness around uterus

Ross (2006) [26]	Number of women randomized: 749<br>Number of women analyzed: group A: 384; group B: 365<br>Age (mean ± SD) (years): group A: 30.1 ± 8.4; group B: 30.5 ± 8.5<br>Number of centres (country): 13 (Denmark; Finland; France; Germany; Greece; Hungary; Italy; Lithuania; Poland; Russia; South Africa; Sweden; UK)	Group A: moxifloxacin 400 mg PO once daily for 14 days.<br><br>Group B: ofloxacin 400 mg PO twice daily + metronidazole 500 mg PO twice daily for 14 days	Primary outcome: clinical cure (5-24 days post-therapy): reduction of the pelvic pain score by > 70% (McCormack score) + apyrexia (rectal/tympanic/oral temperature < 38.0 °C or axillary/cutaneous temperature < 37.5 °C) + WBC count < 10,500/mm^3.<br>Secondary outcomes: microbial cure of *Chlamydia trachomatis*; microbial cure of *Neisseria gonorrhoeae*
Roy (1985) [46]	Number of women randomized: 46 (36 with acute PID)<br>Number of women analyzed: group A: 19; group B: 9; group C: 9<br>Age (years): not stated<br>Number of centres (country): 1 (US)	Group A: cefotaxime 2 g IV or IM every 8 h<br><br>Group B: clindamycin 600 mg IV every 6 h + gentamicin 1.5 mg/kg lean bodyweight IV or IM every 8 h<br><br>Group C: clindamycin 600 mg IV every 6 h + gentamicin 1.5 mg/kg lean bodyweight IV or IM every 8 h + penicillin G 5 million units IM every 4 h	Primary outcomes: clinical cure: antibiotic change was made for treatment failure from an assigned regimen based upon persistence or worsening of signs and symptoms after 48 h
Roy (1990) [47]	Number of women randomized: 67<br>Number of women analyzed: total: 67; group A: 13; group B: 14; group C: 19; group D: 21<br>Age (mean ± SEM) (years): group A: 26.6 ± 1.9; group B: 28.9 ± 1.7; group C: 27.1 ± 1.3; group D: 28.3 ± 1.0<br>Number of centres (country): 1 (US)	Group A: ceftizoxime 2 g IV every 12 h + doxycycline 100 mg IV twice daily<br><br>Group B: ceftizoxime 2 g IV every 6 h + doxycycline 100 mg IV twice daily<br><br>Group C: ceftizoxime 2 g IV every 8 h + doxycycline 100 mg IV twice daily<br><br>Group D: clindamycin 900mg IV every 8 h + gentamicin 2mg/kg loading dose followed by 1.5 mg/kg IV every 8 h with adjustments if necessary	Primary outcomes: clinical cure: adequate response to therapy: clinically improved and afebrile for 48 h at the time of discharge; 8-24 before discharge; no pelvic tenderness; adverse events leading to discontinuation of therapy<br>Secondary outcomes: microbial cure of *Chlamydia trachomatis*, microbial cure of *Neisseria gonorrhoeae*, and length of hospital stay
Savaris (2007) [21]	Number of women randomized: 133<br>Number of women analyzed: group A: 66; group B: 67<br>Age (mean ± SD) (years): group A: 28.3 ± 0.8; group B: 29.27 ± 1.1<br>Number of centres (country): 1 (Brazil)	Group A: ceftriaxone IM 250 mg + azithromycin 1 g PO single dose and repeated after 7 days<br><br>Group B: ceftriaxone IM 250 mg + doxycycline 200 mg PO for 14 days	Primary outcome: clinical cure defined as ≥70% reduction in the total tenderness score at day 14 compared with baseline, for both visual analogue scale and McCormack pain scale<br>Secondary outcome: microbial cure of *Chlamydia trachomatis*

Sirayapiwat (2002) [43]	Number of women randomized: 44; 22 in each group<br>Number of women analyzed: 44; 22 in each group<br>Age (mean ± SD) (years): group A: 31.2 ± 9.1; group B: 24.9 ± 7.6<br>Number of centres (country): 1 (Thailand)	Group A: (triple therapies) received intravenous therapy of 1 gm of ampicillin every 6 hours plus 5 mg/kg. (not exceed 240 mg) of gentamicin once daily and 500 mg of Metronidazole every 8 hours<br><br>Group B: clindamycin 600 mg IV every 8 h + gentamicin 5 mg/kg not exceeding a maximum dose of 240 mg once daily<br><br>In both groups, parenteral therapies continued until women were afebrile for minimum of 48 h then all women received a regimen of doxycycline 100 mg PO every 12 h to complete a 14-day course	Primary outcomes: clinical cure; adverse events leading to discontinuation of therapy<br>Secondary outcome: length of hospital stay. Visual analogue scale on day 3 of treatment
Soper (1988) [48]	Number of women randomized: 62; 31 in each group<br>Number of women analyzed: 62; 31 in each group<br>Age (mean ± SD) (years): group A: 23.4 ± 5.8; group B: 21.9 ± 3.7<br>Number of centres (country): 1 (US)	Group A: cefoxitin 2 g IV every 6 h + doxycycline 100 mg IV every 12 h. Women discharged with doxycycline 100 mg PO twice daily to complete a 10-day course<br><br>Group B: clindamycin 600 mg IV every 6 h + amikacin 7.5 mg/kg IV every 12 h. Women discharged with clindamycin 300 mg PO 4 times daily to complete a 10-day course	Primary outcome: clinical failure: persistence of fever (> 38 °C), elevated WBC (> 11, 000/mm^3), moderate-severe pelvic organ tenderness despite 96 h of antibiotic therapy, need of laparotomy<br>Secondary outcome: length of hospital stay
Sweet (1985) [29]	Number of women randomized: 60; 30 in each group<br>Number of women analyzed: 60; 30 in each group<br>Age (mean ± SD) (years): group A: 24.2 ± 5.1; group B: 24.7 ± 7.2<br>Number of centres (country): 1 (US)	Group A: moxalactam 2 g IV every 8 h<br><br>Group B: clindamycin 600 mg IV every 6 h + tobramycin 1.5 mg/kg every 8 h	Primary outcome: microbiological cure<br>Secondary outcome: length of hospital stay
Thadepalli (1991) [45]	Number of women randomized: 71; group A: 35; group B: 36<br>Number of women analyzed: 61; group A: 31; group B: 30<br>Age (mean (range)) (years): group A: 27.5 (15-37); group B: 26.5 (19-36)<br>Number of centres (country): not stated (not stated)	Group A: ciprofloxacin 300 mg IV twice daily for ≥ 3 days followed by ciprofloxacin 500 mg PO twice daily for about 1 week<br><br>Group B: clindamycin 600 mg IV every 6 h + gentamicin 80 mg IV every 8 h, administered separately. Clindamycin by IV route for 3 days, followed by oral administration for about 1 week. Gentamicin dose adjusted based on serum creatinine and gentamicin levels	Primary outcomes: clinical cure: when there was resolution or clearing (or both) of signs of infection as evidenced by defervescence, reversal of leukocytosis, and abatement of abdominal pain and cervical motion tenderness; adverse events leading to discontinuation of therapy<br>Secondary outcome: length of hospital stay
Tison (1988) [27]	Number of women randomized: 40; 20 in each group<br>Number of women analyzed: 40; 20 in each group	Group A: amoxicillin-clavulanate 1 g slow IV perfusion every 8 h. PO once temperature normal for 48 h, 4 tablets of 500 mg, 2 at a time as long as hospitalized. After discharge, treatment continued with 3 tablets daily of amoxicillin-clavulanate for 3 weeks	Effectiveness: cure: absence of pelvic pain on examination and referred by women, normal body temperature at discharge and 3 weeks later

	Age (mean (range)) (years): group A: 27.5 (17-39); group B: 22.5 (17-34)<br>Number of centres (country): 1 (France)	Group B: penicillinG10million units/24 h IV, followed by penicillinVPO+ gentamicin 160 mg/24 h IM for 10 days + metronidazole 500 mg IV 3 times daily followed by metronidazole 500 mg PO 3 times daily<br><br>Duration of treatment unclear.	Adverse events: any antibiotic-related adverse event leading to discontinuation of therapy during hospital stay and after 30 days
Walters (1990) [32]	Number of women randomized: 147; unclear how many in each group<br>Number of women analyzed: group A: 63; group B: 67. PP analysis only<br>Age (mean ± SD) (years): total age not stated; group A: 25.4 ± 7.7; group B: 24.5 ± 7.8<br>Number of centres (country): 1 (US)	Group A: gentamicin 2.0 mg/kg IV as loading dose, then 1.5 mg/kg IV every 8 h + clindamycin 900 mg IV every 8 h for minimum 4 days. After discharge, clindamycin 450 mg PO every 6 h for total 14 days<br><br>Group B: cefoxitin 2 g IV every 6 h + doxycycline 100 mg IV every 12 h for minimum 4 days. After discharge, doxycycline 100 mg PO every 12 h for total 14 days	Primary outcomes: clinical cure defined as oral temperature < 38 °C for 48 h, resolution of pain and tenderness, and no increase in the size of any pelvic mass after 21 days of initiation of treatment<br>Secondary outcomes: microbiological clearance of *Chlamydia trachomatis*; microbiological clearance of *Neisseria gonorrhoeae* after 21 days of initiation of treatment
Wendel (1991) [22]	Number of women randomized: 96; unclear how many to each group<br>Number of women analyzed: group A: 35; group B: 37. PP analysis only<br>Age (mean) (years): total age not stated; group A: 24.7; group B: 23.3<br>Number of centres (country): 1 (US)	Group A: cefoxitin 2 g IM + probenecid 1 g PO followed by doxycycline 100 mg PO every 12 h for 10 days<br><br>Group B: ofloxacin 400 mg PO every 12 h for 10 days	Primary outcomes: clinical cure defined as complete resolution of tenderness (> 65% decrease in clinical score); adverse events leading to discontinuation of therapy<br>Secondary outcomes: microbiological clearance of *Chlamydia trachomatis*; microbiological clearance of *Neisseria gonorrhoeae*

ESR: erythrocyte sedimentation rate; h: hour; IM: intramuscular; ITT: intention to treat; IUD: intrauterine device; IV: intravenous; n: number of women; PID: pelvic inflammatory disease; PO: per os; PP: per protocol; SD: standard deviation; WBC: white blood cell
